# Four Copies of *SNCA* Responsible for Autosomal Dominant Parkinson's Disease in Two Italian Siblings

**DOI:** 10.1155/2015/546462

**Published:** 2015-11-09

**Authors:** Rosangela Ferese, Nicola Modugno, Rosa Campopiano, Marco Santilli, Stefania Zampatti, Emiliano Giardina, Annamaria Nardone, Diana Postorivo, Francesco Fornai, Giuseppe Novelli, Edoardo Romoli, Stefano Ruggieri, Stefano Gambardella

**Affiliations:** ^1^IRCCS Neuromed, Località Camerelle, 86077 Pozzilli, Italy; ^2^Molecular Genetics Laboratory UILDM, Santa Lucia Foundation, 00142 Rome, Italy; ^3^Department of Biomedicine and Prevention, School of Medicine, University of Rome “Tor Vergata”, 00133 Rome, Italy; ^4^Fondazione Policlinico Tor Vergata, 00133 Rome, Italy; ^5^Department of Translational Research and New Technologies in Medicine and Surgery, University of Pisa, 56126 Pisa, Italy

## Abstract

*Background*. Parkinson's disease (PD) is mostly characterized by alpha-synuclein (*SNCA*) aggregation and loss of nigrostriatal dopamine-containing neurons. In this study a novel* SNCA* multiplication is described in two siblings affected by severe parkinsonism featuring early onset dyskinesia, psychiatric symptoms, and cognitive deterioration.* Methods*.* SNCA *dosage was performed using High-Density Comparative Genomic Hybridization Array (CGH-Array), Multiple Ligation Dependent Probe Amplification (MLPA), and Quantitative PCR (qPCR). Genetic analysis was associated with clinical evaluation.* Results*. Genetic analysis of siblings showed for the first time a 351 Kb triplication containing* SNCA* gene along with 6 exons of* MMRN1* gene in 4q22.1 and a duplication of 1,29 Mb of a genomic region flanking the triplication.* Conclusions*. The identification of this family indicates a novel mechanism of* SNCA* gene multiplication, which confirms the genomic instability in this region and provides data on the genotype-phenotype correlation in PD patients.

## 1. Introduction

Parkinson's disease (PD) is caused by neuronal loss in various monoamine-containing nuclei [1], which typically involve dopamine neurons of the substantia nigra (SN). The analysis of inherited PD allowed identifying a growing number of loci and genes which associate with quite distinct clinical features. Among various genetic abnormalities, a point mutation of* SNCA* (synuclein, alpha [non-A4 component of amyloid precursor]), the gene coding for alpha-synuclein, was the first to be identified in 1997 in the so-called “Contursi family,” carrying an autosomic dominant inherited PD associated with an altered primary structure of alpha-synuclein [2].

Whole* SNCA* multiplications (duplications or triplications) were described later on [3, 4] as responsible for autosomic dominant inherited PD. These patients express extra copies of* SNCA* leading to overexpression of the alpha-synuclein protein owing normal primary structure. Remarkably,* SNCA* is the only gene which produces PD even when it is expressed in a normal structure but in multiple copies, which relates directly to disease onset and severity [5–12].

In fact, increasing* SNCA* copies associates with earlier onset, motor and cognitive dysfunction, psychiatric disturbances, and L-Dopa-induced symptoms [13, 14]. The clinical phenotypes deriving from* SNCA* multiplication often shift towards dementia with Lewy Bodies (DLB) or Frontotemporal Dementia (FTD). In keeping with a dosing effect, while* SNCA* triplication always possesses full penetrance, this may not occur following* SNCA* duplication [15].

Patients with four* SNCA* copies (either following duplication of both alleles or due to a single allele triplication associated with a normal allele) suffer from earlier disease onset and faster progression compared with patients carrying three* SNCA* copies [8, 9, 11, 16, 17].

In the process of establishing specific clinical phenotypes induced by various* SNCA* genotypes in PD, we describe here the effects induced by a novel mutation of* SNCA* which consists of a 351 Kb triplication containing the* SNCA* gene along with 6 exons of* MMRN1* gene in 4q22.1 and a duplication of 1,29 Mb of the genomic region flanking the* SNCA* +* MMRN1* triplication.

## 2. Patients and Methods

### 2.1. Patients

**Figure 1:**
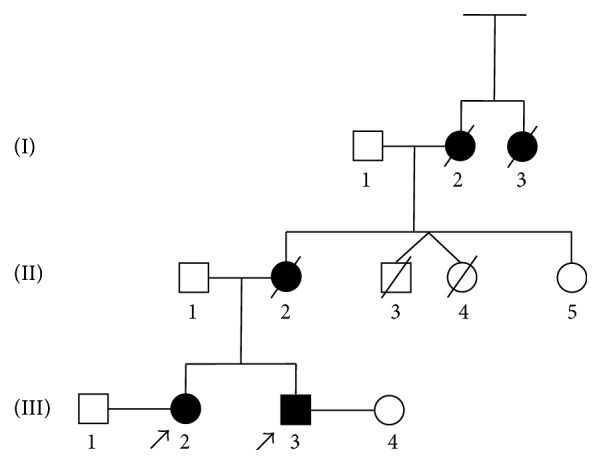
Pedigree of the family of siblings with Parkinson's disease (PD). Black boxes represent affected patients.

The familial pedigree of the family from central Italy shows autosomal dominant pattern of inheritance for PD (Figure 1). The siblings (III;3), a 39-year-old man and his sister (III;2) a 45-year-old woman, are affected by PD. The mother (II;2) and the maternal grandmother (I;2) were affected by PD and died at 43 and 45 years of age, respectively. The patient I;3 was reported to be affected by PD with unknown age at onset. The subjects II;3 and II;4 were unaffected dizygotic twins: II;3 died at birth for delivery complications and II;4 died at old age for cerebral neoplasia. No evidence of PD was reported in the paternal pedigree of patients. For each patient an informed written consent was obtained. This study has been approved by ethical committee.

### 2.2. Genetic Analysis

#### 2.2.1. Multiple Ligation Dependent Probe Amplification (MLPA)

The commercially available kit P051-P052 (MRC-Holland, Amsterdam, Netherlands) was used for the multiplex dosage of exons for the following genes:* TNFRSF9* (1 probe in P051),* DJ1* (4 probes in P051),* ATP13A2* (2 probes in P051, 2 probes in P052),* SNCA* (5 probes in P051, 1 probe in P052),* LPA* (1 probe in P051),* PARKIN* (12 probes in P051, 12 in P052),* LRRK2 *(8 probes in P052),* PINK1* (8 probes in P051),* GCH1* (5 probes in P052),* PACRG* (1 probe in P052),* CAV1/2* (2 probes in P052), and* UCHIL1* (4 probes in P052). The MLPA was performed on DNA from patients III;3 and III;2, and four normal subjects were used as internal controls.

#### 2.2.2. High-Density Comparative Genomic Hybridization Array (CGH-Array)

High-Density Comparative Genomic Hybridization Array (CGH-Array) was carried out using a high resolution whole genome oligo-array (Cytochip Oligo 180 K, BlueGnome, Cambridge, UK).

A sex-matched DNA (Promega, Madison, UK) was used as reference. Digestion, labeling, and hybridization were performed following the manufacturer's protocol (http://www.cytochip.com). Slides were scanned using an Agilent Scanner, with a 5 *µ*m resolution. Data were analyzed using Blue Fuse Multi software (BlueGnome, Cambridge, UK).

#### 2.2.3. Quantitative PCR (qPCR)

qPCR was performed in a CFX ConnectTM Real Time System (Bio-Rad Life Science, CA) using SYBR Green PCR Master (Applied Biosystems, Foster City, CA). Genes included in the triplication (*SNCA*,* MMRN1*), in the duplication (*TIGD2*), and in wild-type (*CCSER1*,* GRID2*, and* PTPN13*) locus were analyzed. The relative copy number was calculated through a ΔΔCT method, using *β*-Globin as an internal reference. qPCR was performed in triplicate for each sample. The reagents used for amplification in 15 *μ*L reactions were 10 *μ*L SYBR Green PCR Master (Applied Biosystems, Foster City, CA), 0.5 *μ*M of each primer, and 5 ng of genomic DNA. The PCR conditions were 95°C for 10 min, 95°C for 30 s, and 58°C for 1 m (40 cycles).

## 3. Results

### 3.1. Patient III;3

Patients III;3 came to our attention in 2011 with a hypokinetic-rigid syndrome prominent on the left side. The Unified Parkinson's Disease Rating Scale (UPDRS) III score was 35 in “off” and 14 in “on,” Hoehn and Yahr scale was 3, and motor fluctuations and some slight axial dyskinesia appeared. III;3 also presented moderate camptocormia, kinetic ataxia, and moderate dysarthria that were more evident in “off” state. During anamnesis patient reported the diagnosis of PD back in 2003 (at 28 years of age) and he reported a rapid motor deterioration; he also complained of depressed mood and altered sleep time. When we evaluated the patient (2011), he was under treatment with a dopamine agonist (rotigotine) and L-Dopa (400 mg/day).

Due to the presence of symptoms reported above, therapy was changed by fractioning the daily L-Dopa administration by using L-Dopa/carbidopa/entacapone 50/12.5/200 mg 5 times per day; we maintained rotigotine (6 mg per day) and added rasagiline (1 mg per day). A few months later he developed visual hallucinations, delusion of jealousy, and aggressive behaviors. Therefore, we withdrew the dopamine agonist rotigotine and we modified L-Dopa administration switching from L-Dopa/carbidopa/entacapone 50/12.5/200 mg 5 times per day to L-Dopa/carbidopa 100/25 4 times per day therapy. We kept rasagiline (1 mg per day) while adding clozapine (50 mg/day) to treat psychiatric symptoms which indeed disappeared in few weeks.

In the years 2012-2013, there was a progression of UPDRS III score which increased (UPDRS III 42 in “off” and 23 in “on state”). At this point, camptocormia and standing and walking difficulties were evident with mild trunk bending (Pisa syndrome). Also nonmotor symptoms worsened; cognitive impairment was evident and became very severe: Mini-Mental State Examination (MMSE) score was 14/30 in 2012 whereas in 2014 the scale was not applicable anymore because the patient was unable to understand the questions and he was unable to speak fluently. Cognitive deficits at this time were severe in all domains and patients lost his autonomy in daily life. At this time we could document REM sleep disorder, dysphagia, and urinary incontinence. In a few months all motor and nonmotor symptoms further deteriorated; thus, standing and walking were impossible and dysarthria, cognitive decline, and incontinence were very severe and led soon after to the loss of autonomy in each daily activity. In the evaluation of July 2015, Hoehn and Yahr score was 4 and UPDRS was >60 in “off” and 42 in “on” state. Therapy was not modified consisting mainly in L-Dopa and clozapine. Patient is on a wheelchair or in bed. Dysphagia is severe and nasogastric tube is necessary for hydration.

PET with fludeoxyglucose (FDG) shows frontotemporal and parietal degeneration. In 2011 magnetic resonance imaging (MRI) showed slight frontotemporal atrophia with some subcortical gliotic spots. In 2015 the MRI showed severe cortical and subcortical atrophia in temporoinsular and frontal regions bilaterally. Thus, brain atrophy at MRI worsened within frontal regions and mostly within temporal regions, bilaterally.

### 3.2. Patient III;2

Patient III;2, sister of III;3, was referred to us in 2012 at the age of 43 years reporting a diagnosis of PD dating back to 2011 year. She suffered from a mild hypokinetic-rigid syndrome on the right side. Hoehn and Yahr score was 2 and UPDRS III was 16 in “off” and 7 in “on” with a significant response to L-Dopa. Motor symptoms deteriorated but response to L-Dopa was still present. The PD score in 2015 was as follows: UPDRS III 30 in “off” and 15 in “on” and Hoehn and Yahr scale 3. She did not have cognitive decline (MMSE was 28/30 in 2011-2012), although in 2014 she worsened (MMSE 24/30). At this time cognitive functions were impaired for attention and visuospatial memory, and she also developed slight motor apraxia. She reported REM sleep behavior disorders and depressive episodes, but she refused a psychiatric evaluation. She is under therapy with L-Dopa/Carbidopa 100/25 four times per day.

MRI carried out in 2012 shows mild pontocerebellar degeneration and slight millimetric gliotic spots. SPECT DatSCAN shows bilaterally reduced binding in the putamen, more pronounced on the left side.

### 3.3. Genetic Analysis

**Figure 2:**
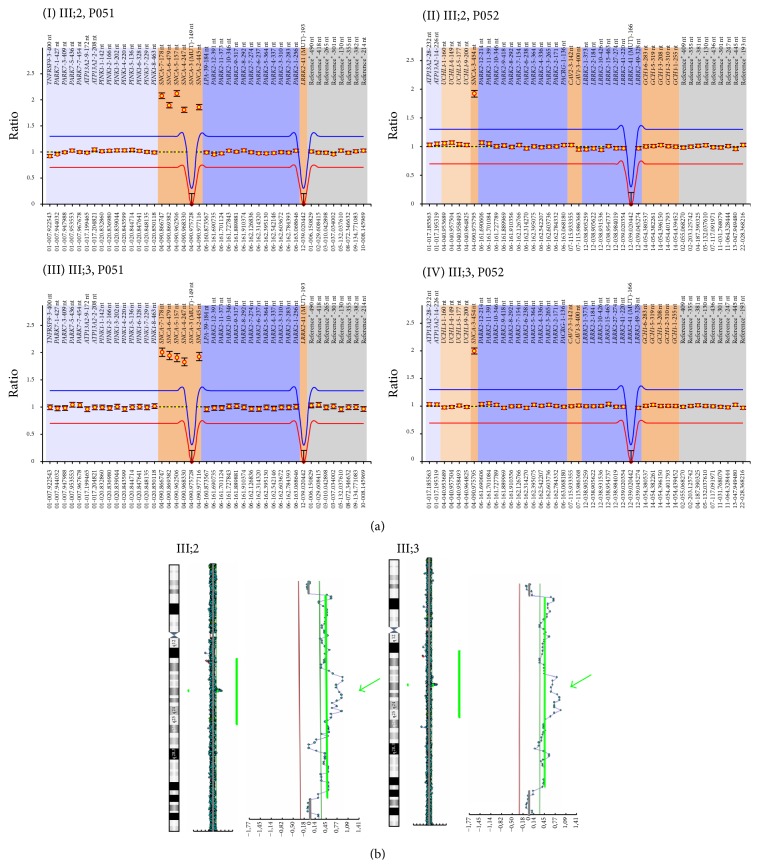
*SNCA* dosage using CGH-Array and MLPA. (a) MLPA analysis. In these images, normalized peak areas relative to probes considered in MLPA kit P051 (I and III, resp., III;2 and III;3) and P052 (II and IV, resp., III;2 and III;3) are shown. (I and III) The probes related to* TNFRSF9* (one probe),* PARK7* (twelve probes), and* ATP13A2* (two probes) genes are shown in light blue,* SNCA* (five probes) and two mutation specific probes for p.A30P in* SNCA* and p.G2019S in* LRRK2* genes in brown,* LPA* (one probe) and* PARK2* (twelve probes) genes in dark blue, and the reference probes (eight) in gray. (II and IV) The probes related to genes* ATP13A2* (two probes) and* UCHL1* (four probes) are shown in light blue,* PARK2* (twelve probes), PACRG (one probe),* LRRK2* (eight probes), and one mutation specific probe for p.G2019S in* LRRK2* in dark blue,* SNCA* (one probe),* CAV1* (one probe),* CAV2* (one probe), and* GCH1* (five probes) in brown, and the reference probes (nine) in gray. In both patients, an increased ratio in the peak area related to* SNCA* probes indicates a multiplication at 4q21 chromosomal region. In particular, ratio of* SNCA* probes in III;2 (*SNCA* ex2 ratio 1.86,* SNCA* ex4 ratio 1.81,* SNCA* ex5 ratio 2.12,* SNCA* ex6 ratio 1.89,* SNCA* ex7 ratio 2.08, and* SNCA* ex3 ratio 1.92) and III;3 (*SNCA* ex2 ratio 1.92,* SNCA* ex4 ratio 1.83,* SNCA* ex5 ratio 1.91,* SNCA* ex6 ratio 1.95,* SNCA* ex7 ratio 2.01, and* SNCA* ex3 ratio 2) suggests the presence of four copies of* SNCA* locus. (b) Array-CGH profile of chromosome 4 showing a duplication/triplication at 4q21 from 90.013 Mb to 91.26 Mb. Enlargements indicate the duplicated region, and green arrows show the triplicated region. For each analysis, *Y*-axis marks the distance along chromosome 4, and *X*-axis marks the hybridization ratio plotted on a log2 scale. Red lines indicate thresholds for clone deletion and green lines for duplication.

MLPA analysis revealed the presence of four copies of* SNCA* in both III;2 and III;3 (Figure 2(a)). To characterize the size of multiplicated genomic segment, we performed CGH-Array that revealed, in both III;2 and III;3, three copies of the region at 4q22.1 ranging from 90,013,153 Mb to 91,310,633 Mb (build36, hg18, involved genes are* TIGD2* (MIM^*∗*^612973),* GPRIN3* (MIM^*∗*^611241),* SNCA* (MIM^*∗*^163890),* MMRN1* (*Multimerin 1*, MIM^*∗*^601456), and the first three exons of* CCSER1 *(*FAM190A*)) and four copies of the region ranging from 90,500,031 Mb to 90,851,296 Mb containing* SNCA* gene and 6 exons of* MMRN1* (Figure 2(b)).

To confirm the right dosage detected with CGH-Array, we performed qPCR analysis of the 3 genes involved in the multiplicated region (*SNCA*,* TIGD2,* and* CCSER1*) and 2 control genes located in 4q22 not involved in the multiplication (*GRID2* (human glutamate receptor delta 2) and PTPN13 (protein tyrosine phosphatase, nonreceptor type 13)). We detected the presence of four copies of* SNCA*, three copies of* TIGD2*, and two copies of* CCSER1* (probe on exon 11 outside of duplication),* GRID2,* and* PTPN13*, confirming the results obtained by CGH-Array.

**Figure 3:**
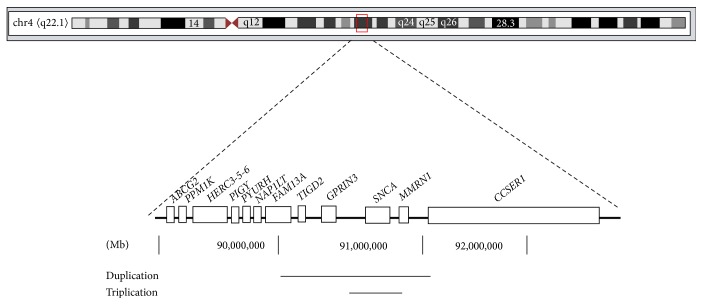
Schematic representation of the multiplication described on chromosome 4, consisting of a 351 Kb triplication of a locus containing* SNCA* and 6 exons of* MMRN1* gene (90.500.031 Mb to 90.851.296 Mb) and a duplication of 1,29 Mb of a genomic region flanking the triplication, involving* TIGD2*,* GPRIN3*,* SNCA*, and* MMRN1* and from exon 1 to exon 3 of* CCSER1* (90.013.153 Mb to 91.310.633 Mb). Scale is expressed in Megabases (Mb).

Patients' mother (II;2) and maternal grandmother (I;2) were both affected by PD and died at ages 43 and 45, respectively, and no history of PD was recorded in the paternal ancestors. Thus, assuming the absence of inherited parkinsonism-related mutations in the paternal ancestors, the present data indicate* in*-*cis* mechanism for this mutation, with a 351 Kb triplication of locus containing* SNCA* and 6 exons of* MMRN1* gene and a duplication of 1,29 Mb of a genomic region flanking the triplication, involving* TIGD2*,* GPRIN3*,* SNCA*, and* MMRN1* and from exon 1 to exon 3 of* CCSER1* (Figure 3).

## 4. Discussion

In this study, we briefly report for the first time a multiplication of* SNCA* characterized by four copies of* SNCA* three of which derive from a triplicated region of 351 Kb containing* SNCA*, described with a duplication of 1,29 Mb of a genomic region flanking the triplication. Quantitative analysis performed in this paper could not provide useful information about the genome location of the triplication and duplication identified in this family.

To date, only 7 families have been reported bearing four copies of* SNCA*. Among these, 6 families have a triplication, and a family has a homozygote duplication of* SNCA* locus [4, 17–23].

The multiplication detected in our family consists of a triplication of 351 Kb, thus shorter than the triplications previously described in the Iowa family (1,6–2,1 Mb), Lister Branch I (0,9 Mb) from USA, and FPD-014 from France (2,64 Mb) [4, 17, 18]. The size of the triplication is not exactly specified in the paper by Keyser and in the Asian family reported by Sekine, the range of triplication includes whole exons of* SNCA* and* MMRN1* and exon 1 of* KIAA1680* probably (90,645,250–90,759,447) [19, 21]. Different duplication size has been reported so far, ranging from 400 Kb in family A from Japan up to more than 4 Mb in family FPD-131 from France [8–10, 17, 24–26].

The specificity of the genetic pedigree we describe here consists in a* SNCA* triplication which also includes the six exons of* Multimerin 1* (*MMRN1*).* MMRN1* encodes for a specific Factor V/Va binding protein found in platelets and endothelium [23].* MMRN1* deficiency is associated with Factor V Quebec, an inherited bleeding disorder, although the consequences of its overexpression remain unknown.* MMRN1* is not expressed in brain; thus, wild-type overexpression of alpha-synuclein appears responsible for the predominant, neurological phenotype. So far, functional studies of brain tissue from PD patients with* SNCA* multiplications indicate that disease conditions depend directly on the number of copies of* SNCA*, while the concomitant multiplication of genes contiguous to* SNCA* does not play any effect [18]. However, there is lack of specific investigation on the potential role of* MMRN1* which is now under analysis in our institute.

The* SNCA* multiplication described here for the first time is associated with severe PD symptoms in siblings and maternal ancestors.

According to previous reports on* SNCA* multiplications, clinical phenotype of III;2 and III;3 consists of severe parkinsonism. This confirms the genotype-phenotype correlations for* SNCA* reported so far [5–12]. In particular, these siblings are characterized by early onset, rapid progression up to motor deterioration, cognitive and psychiatric symptoms, and sleep disturbances. Imaging confirmed the occurrence of brain alterations involving multiple areas beyond basal ganglia and mostly involving frontoparietal regions, thus strengthening the complex clinical phenotypes of these patients.

## 5. Conclusion

We briefly report severe parkinsonism associated with a novel multiplication in the* SNCA* gene which confirms a deleterious correlation between expression of* SNCA* gene and disease severity. This is likely to depend on* SNCA*-induced dose-dependent toxicity. The uniqueness of* SNCA* multiplication consists in the onset of parkinsonism associated with a normally structured protein expressed in abnormal amount. This opens new avenues both to genetic and to environmental factors and to how they interact in PD. In fact, in the process of redefining PD [27], cell to cell disease spreading of alpha-synuclein is likely to drive the amount of affected brain regions which in turn relates to the number of nonmotor symptoms [28, 29]. In keeping with this, the spreading of alpha-synuclein appears as a key pathogenic mechanism in sporadic PD. Thus, a special emphasis should be devoted to those environmental agents which may regulate a variety of multiplications of* SNCA*.
